# Accuracy of Coronal Plane Mechanical Alignment in a Customized, Individually Made Total Knee Replacement with Patient-Specific Instrumentation

**DOI:** 10.1055/s-0037-1608946

**Published:** 2017-12-14

**Authors:** Gary A. Levengood, Jack Dupee

**Affiliations:** 1 Department of Orthopaedics, Sports Medicine South, Lawrenceville, Georgia

**Keywords:** customized knee implant, mechanical alignment, patient-specific instruments, computer-assisted surgery, total knee arthroplasty

## Abstract

The objective of this study was to evaluate the accuracy of a customized individually made total knee implant used in conjunction with patient-specific cutting guides in restoring coronal plane mechanical axis alignment using computer-assisted surgery (CAS). A consecutive series of 63 total knee arthroplasty (TKA) patients were prospectively measured with intraoperative CAS. The patient-specific instruments and implants were created utilizing a preoperative CT scan. CAS system was used for all patients, to determine mechanical alignment. Bone cuts were made using the patient-specific instruments. Both bone cuts and final coronal mechanical alignment were recorded utilizing the navigation system for the assessment.

The patient-specific instruments and implants provided perfect neutral coronal mechanical alignment (0°) in 53 patients. The remaining 10 patients had a postoperative alignment within ± 2° of neutral. The average preoperative deformity was 5.57° versus 0.18° postoperatively ( *p* < 0.0001). The mean correction angle was 5.68°. No patients had postoperative extension deficits as measured with CAS (7.50° pre-op for 40/63 patients). Customized, individually made total knee implant with patient-specific cutting jigs showed results that are comparable to those of CAS systems in this study. This technology restores the neutral coronal mechanical axis very accurately, while offering unique benefits such as improved implant fit and restoration of the patient's J-curves, which require further investigation.

Total knee arthroplasty (TKA) is a standard of care to relieve pain, restore function, and provide overall satisfaction to patients affected by arthritis of the knee joint. 1 Research has shown good mid- to- long-term survivorship following TKA. However, with the increasing elderly population combined with the trend of TKA being conducted on younger, more active patients, there has been an increased effort to provide better clinical outcomes and longer survivorship.

A mechanically neutral limb alignment has been linked to success and increased survivorship post-TKA. 2 3 4 The correct lower limb alignment postsurgery depends on the accurate alignment of the femoral and tibial implant components with respect to existing bone. 5 Previous research 5 6 conducted on the effects of improper limb alignment suggests that malalignment may affect implant function and lead to decreased survival in TKAs. This is due to off-axis loading, polyethylene wear, and subsequent implant loosening. 7 Achieving a normal mechanical axis alignment postsurgery with an alignment of +/− 3° of the mechanical axis reduces the risks of abnormal wear, premature loosening, and early implant failure. 8 9 10

Patient-specific cutting guides have been introduced to provide more accurate bone cuts and better implant alignment without the use of computer-assisted surgery (CAS). The ability of patient-specific cutting blocks to accurately achieve neutral alignment in the coronal plane has previously been reported by multiple studies with varying results. 1 11 12 13 14 15 16 Most of these studies have shown an average alignment that is very close to neutral, but have also found a varying number of outliers (>3°) within specific brands (Otismed, Visionaire, Signature, etc.) as well as between brands.

The objective of this study was to evaluate the accuracy of a customized individually made total knee implant used in conjunction with patient-specific cutting guides in restoring mechanical axis alignment using CAS. We also compared the results with previously published literature. We looked at four parameters: (1) femoral varus/valgus cut angle made by using the iJigs, (2) tibial varus/valgus cut angle made by using the iJigs, (3) mechanical axis alignment of the lower limb postsurgery, and (4) extension deficit pre- to postsurgery and the ability to achieve full leg extension. Our hypothesis was that postoperative mechanical alignment for all patients should be within +/− 3° of neutral.

## Materials and Methods

A consecutive series of 63 patients undergoing TKA, using patient-specific instruments and implants (ConforMIS Inc., Billerica, MA), were recruited for this single surgeon (GL) study. All surgeries were performed at the Gwinnett Medical Center (Duluth, GA) between September 2011 and November 2013. The 63 patients enrolled represented the first 63 surgeries performed utilizing the customized implant and jig system by the lead author. Hospital ethics committee waiver was obtained from the hospital prior to submission.

The average age of the patient cohort was 64.7 years (range 44–89; std. dev.: 10.7) and consisted of 27 males (42.9%) and 36 females (57.1%). Twenty-seven patients were implanted with a right TKA (42.9%), while 36 patients received a left TKA (57.1%). None of the patient cohort received simultaneous bilateral TKAs at surgery and all patients exhibited <15° of varus/valgus deformity prior to surgery. A statistical analysis was not performed on the patient cohort to match sex and surgery side since this was a consecutive patient series and each patient was scheduled for surgery depending on time of first consultation.

A preoperative CT scan was obtained from each patient prior to surgery in accordance with the acquisition protocol of the implant manufacturer. Customized femoral and tibial components were designed using an automated proprietary software (iFit, ConforMIS Inc., Billerica, MA) along with the customized jig set for all bony cuts. This software plans the placement of the TKA components to correct for deformity and place the knee in a neutral mechanical axis alignment postsurgery.

Surgery was conducted for all patients using CAS (AESCULAP Orthopilot TKA v4.3 system, Center Valley, PA) as per the standard operating technique recommended by the manufacturer. Once the knee was prepared, arthrotomy was performed to access the affected joint. Intraoperative registration was performed with bicortical screw trackers in the femur and tibia. Center of rotation of the hip, knee, and ankle was obtained dynamically. Tibial plateau, femoral condyles, medial and lateral epicondyles, medial and lateral malleolus, and tibial spine were then registered according to protocol. The CAS was then used to measure the preoperative mechanical alignment of the lower limb as well as the extension deficit, if present.

The patient-specific instruments were then utilized per the manufacturer's recommendations in the preoperative surgical plan and included the following steps. The tibia was prepared using a custom tibial cutting guide and the extramedullary alignment rod was connected to the patient-specific jig. The tibial cut was made using a posterior cruciate ligament (PCL) preserving approach. With the help of femoral cutting and alignment jigs, the distal femoral cut was performed. The femur was then prepared using four additional customized femoral jigs. Anterior and posterior femoral resections were performed along with three additional chamfer cuts. Once the knee was balanced in flexion and extension, trialing and cementing of the patient-specific implant components were completed. All bone cuts were recorded utilizing CAS as a confirmatory measurement. Final mechanical alignment and extension lag were recorded, again utilizing the CAS to obtain the measurements. It is important to note that CAS was utilized for measurement purposes only, not to determine bone cuts or to modify implant placement.

### Statistical Analysis

To determine the significance between data in each group, a statistical analysis was performed either by using inbuilt or custom functions in Microsoft Excel 2010 (Microsoft Corp, Redmond, WA). Initially, all datasets (pre-op versus post-op) for each comparison criteria (mechanical alignment and extension deficit) were tested for normality using the Anderson Darling Normality Test. Then, for each comparison criteria, a two-tailed student's *t* -test was conducted to determine significance ( *p* < 0.05).

## Results

All surgeries were successfully completed with the use of patient-specific jigs and the customized TKR. There were no complications related to the use of the CAS system, for example, pin fractures.

### Preimplantation Alignment

The average preoperative deformity from neutral, which we defined as a hip-knee-ankle (HKA) angle of 180°, for the entire group was 5.57° (range: 12° valgus to 15° varus; std. dev.: 6.5) ( Table 1 ). This included 4 (6.3%) patients with neutral mechanical axis alignment, 37 patients (58.7%) with a varus deformity (avg: 6.27°, range: 1–15 ^o^ , std. dev.: 3.41°), and 22 patients (34.9%) exhibited a valgus deformity (avg: -5.68°, range: -2 to -12°, std. dev.: 3.27°). Extension deficit was seen in 40 patients (63.5%) with an average of 7.50° (range: 1–30°; std. dev.: 5.58°), while 18 (28.6%) patients exhibited preoperative hyper-extension with an average of -5.44° (range: 1–13°; std. dev.:3.79°). The remaining five patients exhibited a full extension at examination.

**Table 1:** Limb alignment pre- and postoperative

	Preoperative			Postoperative		
	Average	Range	Std. Dev.	Average	Range	Std. Dev.
Limb deformity (HKA)	5.57°	12° valgus to 15° varus	6.5°	0.18°	0° valgus to 2° varus	0.42°

Abbreviations: HKA, hip-knee-ankle; Std. Dev., standard deviation.

### Postimplantation Alignment

Both tibial and femoral alignment were defined as neutral, if the proximal and distal placement, respectively, achieved the target of 90°, that is, were perpendicular to the HKA. The patient-specific instruments and implants provided neutral alignment of the tibial component in 55 of 63 (87.3%) patients, with the remaining 8 (12.7%) patients being within +/− 1° of neutral. A neutral femoral varus/valgus angle was achieved in 48 of 63(76.2%) of the patients with 12 of the remaining 15 patients being within +/− 2°. None of the patients required a recut of the femur or the tibia. Neutral mechanical limb alignment after implantation of all components was seen in 53 of 63 patients (84.1%). Each of the 10 remaining patients had a postoperative alignment within ± 2° of neutral, with no outliers ( Table 2 ). The average postoperative amount of deformity for this cohort was 0.18° (range 0–2°; std. dev.: 0.42) which was found to be statistically significantly different from the preoperative condition ( *p* < 0.0001). The mean correction angle for this cohort was 5.68° (range: 15–0°, std. dev.: 3.48).

**Table 2:** Percentage of patients achieving neutral alignment postoperative

	Postoperative	
	Neutral alignment (%)	±2° of neutral (%)
Tibial component angle	87.3	12.7
Femoral component angle	76.2	19.0
HKA angle	84.1	15.9

Abbreviation: HKA, hip-knee-ankle.

### Extension Deficit

Before implantation, an average extension deficit of 7.50° was observed in 40 of 63 (63.5%) patients. After implantation, none of the 63 patients (100%) exhibited an extension deficit as measured with CAS.

## Discussion

This is one of the first studies, to our knowledge, that has investigated with the use of CAS, the ability of a customized, individually made total knee implant with patient-specific cutting guides to accurately achieve neutral mechanical axis alignment postoperatively. Restoration of the mechanical axis of the lower limb post-TKA has been shown to be a contributing factor in ensuring implant longevity. 2 4 5 6 Previous literature has shown that a mechanical axis alignment >3° from neutral is associated with increased risk of implant failure. 6 This is due to the fact that a poorly aligned knee is not capable of providing balanced loading to the lateral and medial compartments of the polyethylene insert, leading to an unequal force distribution and ultimately failure of the tibial component due to excessive polyethylene wear. A study conducted by Green et al found that there is a definite association between tibial component collapse and tibial varus alignment. 4 Another study conducted by Fang et al on 6070 knees with 51 failures found that there is a statistical, as well as clinical, correlation between implant failure and mechanical alignment. 17

Research conducted to measure mechanical axis alignment postsurgery has found a varying pattern in percentage of patients within +/− 3° from neutral alignment. Lustig et al investigated the accuracy of the Visionaire system on 45 TKAs and looked at the coronal axis postsurgery for the femur, tibia, and the total lower limb. They concluded that the error in femoral alignment from planned alignment was on average -0.2°, the tibial alignment from planned was 0.6°, and the total alignment from planned was 0.6°. However, 20.7% of their subjects experienced an overall error in mechanical axis alignment >3° from neutral. 11 When the threshold was set at 2° from neutral, they found that 44.8% of their knees were virtual outliers.

A study conducted by Ng et al on a series of 569 knees measured after TKA using the Signature patient-specific cutting guides found that 14.4% of patients exhibited a deviation of 3° from neutral. 18 Nunley et al compared the suggested mechanical alignment using two different patient-specific systems and found similar results with the Signature system (18% over 3°), but reported a much higher deviation in the OtisMed system (44%). 15 16

Based on our review of the published literature, these results observed with the customized, individually made total knee implant with patient-specific cutting jigs were found to be more consistently accurate than previous reports on patient-specific cutting guides. Our results suggest that these implants used with patient-specific cutting guides can consistently provide a neutral distal femoral and proximal tibial bony cut, while restoring the mechanical axis. Our hypothesis that all patients will be within +/− 3° from neutral was validated, with 84.1% of the patient cohort exhibiting a 180° HKA neutral mechanical alignment, with no outliers more than +/− 2° from neutral as measured by CAS. Average preoperative extension lag of 7.5° was also corrected effectively during surgery, with none of the 63 patients in the cohort exhibiting extension deficits postsurgery.

We believe there are key differences that may have contributed to the greater accuracy of the iTotal system with its iJig instrumentation compared with the published literature. All other patient-specific cutting jig systems consist of a single starter jig on the femur and a single starter jig on the tibia. These jigs are used to guide pin placement for attaching standard cut blocks or for placing a first cut. All subsequent steps utilize standard instrumentation. The use of a full set of patient-specific jigs with the iTotal implant, with each jig customized to the patient, may result in greater accuracy. Importantly, the iJig system is designed to provide 90° cuts of the distal femur and proximal tibia in relation to the neutral HKA. This allows the native medial and lateral offsets of the femur to be maintained in the customized implant design and matched on the tibial side with differing insert thicknesses medially and laterally. This obviates the need to introduce varus or valgus cuts on the femur and tibia to maintain the proper joint angle and achieve a neutral HKA angle.

This combination of factors may contribute to the higher accuracy of the iTotal implant system in the present study. Similar accuracy in alignment has been recently reported in a study reporting on a customized partial knee system using patient-specific jig instrumentation as well as customized total knee systems. Koeck et al used full leg weight-bearing standing X-rays for the pre- and postoperative alignment assessment. 19 Similarly, using weight-bearing X-rays for measurement purposes, Ivie et al reported that patients with a patient-specific TKA were 1.8 times more likely to achieve proper alignment when compared with conventional TKAs. 20

Use of a CAS as the reference for the measurements of the mechanical axis pre- and postimplantation could be one of the limitations of this study. The measurements arising from CAS are dependent on what is registered and data may be incorrect if the original registration is not accurate. However, CAS has been commonly used during surgery for aligning implant components. Also, the lead author is trained in using CAS and has used them in >600 surgeries prior to use in this study. We believe that this has a mitigating impact on registration errors. Additionally, CAS systems have been found to be more accurate than radiographic and CT measurements and prevent the patient from being exposed to additional ionizing radiation. 21 22

Another limitation of this study is the fact that this study was conducted on a sample size that is smaller than the average volume of an orthopedic surgeon in the time window analyzed, though it is comparable to similar previously published studies. This could be seen as a limiting factor in powering the study. Nevertheless, these were consecutively recruited patients at a sports medicine practice and the results of this study indicate that the results are highly reproducible; therefore, we do not anticipate a deviation from the current results by increasing the sample size. Also, the sample size used for this study is comparable to previously published reports on mechanical alignment using CAS. 11

As part of the study data collection, sagittal plane alignment of the femoral and tibial bones, pre- and postimplantation was not collected. Presurgery femoral and tibial varus/valgus alignment was not assessed. The goal of our study was to evaluate the iJig system used in conjunction with the iTotal implant in reproducing overall coronal plane mechanical alignment after implantation and the ability of the system to return the patients to full extension. These data have been presented in the study. Future studies that investigate the sagittal alignment in conjunction with the coronal alignment using these jigs will provide a deeper understanding on the ability of the iJig system to restore sagittal and coronal alignment postsurgery.

Lastly, the study does not include a control group, which would have provided a direct comparison of outcomes. There are multiple studies that have investigated the use of patient specific instrumentation blocks in conjunction with off-the-shelf implants. We believe comparing our results to the results presented in these manuscripts as an adequate criterion for comparing the outcomes with the customized implant used with the iJigs platform. It is important to note, however, that many of these studies investigated the use of patient-specific jigs manufactured using MRI imaging. The patient-specific jigs investigated in this study are manufactured using CT imaging. The differences in the imaging modalities were not investigated in this study.

In conclusion, the customized, individually made total knee implant with patient-specific cutting jigs showed results that are comparable to those of CAS in this study. The technology restores the neutral mechanical axis very accurately and results were favorable when compared with previous studies investigating patient-specific starter jigs used in conjunction with standard, off-the-shelf implants.
